# Influences of the Thomson Effect on the Performance of a Thermoelectric Generator-Driven Thermoelectric Heat Pump Combined Device

**DOI:** 10.3390/e20010029

**Published:** 2018-01-05

**Authors:** Yuanli Feng, Lingen Chen, Fankai Meng, Fengrui Sun

**Affiliations:** 1 Institute of Thermal Science and Power Engineering, Naval University of Engineering, Wuhan 430033, China; 2 Military Key Laboratory for Naval Ship Power Engineering, Naval University of Engineering, Wuhan 430033, China; 3 College of Power Engineering, Naval University of Engineering, Wuhan 430033, China

**Keywords:** thermoelectric generator, thermoelectric heat pump, combined thermoelectric device, Thomson effect, non-equilibrium thermodynamics, exergy analysis, temperature dependence, performance analysis and optimization

## Abstract

A thermodynamic model of a thermoelectric generator-driven thermoelectric heat pump (TEG-TEH) combined device is established considering the Thomson effect and the temperature dependence of the thermoelectric properties based on non-equilibrium thermodynamics. Energy analysis and exergy analysis are performed. New expressions for heating load, maximum working temperature difference, coefficient of performance (COP), and exergy efficiency are obtained. The performance is analyzed and optimized using numerical calculations. The general performance, optimal performance, optimum variables, optimal performance ranges, and optimum variable ranges are obtained. The results show that the Thomson effect decreases the general performance and optimal performance, and narrows the optimal operating ranges and optimum variable ranges. Considering the Thomson effect, more thermoelectric elements should be allocated to the thermoelectric generator when designing the devices. The optimum design variables for the maximum exergy efficiency are different from those for the maximum COP. The results can provide more scientific guidelines for designing TEG-TEH devices.

## 1. Introduction

The thermoelectric (TE) effect [1] can be used to directly convert between heat and electricity. It is the most effective way to realize power generation, cooling, and heating with zero emission [2], and to utilize low-grade heat and to recycle waste heat [3]. Traditional TE devices [4] are categorized into TE generator (TEG), TE heat pump (TEH), and TE cooler (TEC). Combined TE devices, which are TEC- or TEH-driven by a TEG, are a new type of TE device. Due to its advantages of environmental protection, high reliability, no working medium, no extra power supply, and thermal insulation and saving on piping material, combined TE devices have unique values and potential for applications [5].

Non-equilibrium thermodynamic theory [6,7,8] is the basic theory to analyze the performance of TE devices. Some researchers have studied TEGs [9,10,11,12], TEHs [13,14,15], TECs [16,17,18,19], and combined TE devices [20,21,22,23] based on non-equilibrium thermodynamics, and some meaningful results have been gained. Vella et al. [20] proposed a TEG-driven TEC (TEG-TEC) combined device that uses solar energy as the heat source. Meng et al. [21,22,23] proposed a TEG-driven TEH (TEG-TEH) combined device, analyzed the performance, optimized the allocation of the TE element [21], investigated the maximum heating temperature difference of the TEG-TEH device [22], and studied the effect of physical size of the TE element on the performance of the TEG-TEH device [23].

The Thomson effect [24,25], which describes the heating or cooling when electrical current flows through a conductor with a temperature gradient, is one of the TE effects. The Thomson effect has been neglected in most thermodynamic studies on TE devices [9,10,11,12,13,14,15,16,17,18,19,20,21,22,23]. However, some researchers have found that the Thomson effect has a notable influence on the performance of TE devices. Chen et al. [26,27] studied the Thomson heat in TEGs [26] and TEHs [27] and pointed out that the Thomson effect should be considered in thermodynamic studies of TE devices. Manikandan and Kaushik [28,29] and Lamba and Kaushik [30] investigated the influence of the Thomson effect on the performances of TEGs [28], TEHs [29] and TECs [30], and the results have indicated that the Thomson effect decreases the performances of TEGs and TEHs, and increases the performance of TECs. Ortega and Olivares-Robles [31] studied the Thomson heat in two kinds of two-stage TECs with the same and different TE materials and found that the Thomson effect enhances performance slightly. Feng et al. [32] studied the performance of a TEG-TEC combined device and the results indicate that the Thomson effect decreases the cooling capacity, coefficient of performance (COP), and maximum cooling temperature difference.

The exergy analytical method [33,34] is used to evaluate thermodynamic systems from the perspectives of quantity and quality, combining the first law with the second law of thermodynamics. Exergy efficiency [35,36], which is used to measure the efficiency of energy conversion of a thermodynamic system, is more comprehensive than the first law efficiency. Exergy losses can be pinpointed by means of an exergy analysis, so as to reduce the avoidable exergy losses by taking proper measures [37,38,39,40]. Sharma et al. [41] performed exergy analysis for single and two-stage TECs and studied the effect of allocation of the TE element on exergy efficiency and exergy loss for two-stage TECs. Islam et al. [42] performed exergy analysis for TEGs driven by solar energy. Nemati et al. [43] optimized the length of the TE element of TEHs for maximum exergy and exergoeconomic performance. Manikandan and Kaushik [28,29] and Lamba and Kaushik [30] carried out detailed energy analysis and exergy analysis for TEGs [28], TEHs [29], and TECs [30], and studied the effect of shape parameters on exergy efficiencies.

The Seebeck coefficient, electrical resistance, thermal conductivity, and the Thomson coefficient of TE materials are temperature dependent. TE devices have been studied in the case of constant TE properties in many relevant literature in order to simplify the calculation process [9,10,11,12,13,14,15,16,17,18,19,20,21,22,23]. Some researchers have performed thermodynamic studies on traditional TE devices in the case of temperature-dependent TE properties [28,29,30,44,45,46,47,48], and some practical results have been gained. Manikandan and Kaushik [28,29] and Lamba and Kaushik [30] calculated the Seebeck coefficient, electrical resistance, thermal conductivity, and the Thomson coefficient of TEGs [28], TEHs [29], and TECs [30] by fitting formulas. Meng et al. [44] found that the temperature dependence of TE properties leads to more accurate calculated power output and thermal efficiency of TEGs. Feng et al. [32] studied the TEG-TEC combined device with considering the temperature dependence of TE properties.

However, the influence of the Thomson effect on the performance of the TEG-TEH combined device has not been investigated so far. A non-equilibrium thermodynamic model of the TEG-TEH combined device, which considers the Thomson effect and the temperature dependence of TE properties, will be established in this paper using methods in References [28,29,30] on the basis of studies in References [21,22,23]. The performance characteristics will be analyzed. The allocation of TE elements will be optimized for a fixed total number of TE elements. The difference between the optimization results for the maximum exergy efficiency and for the maximum COP will be investigated. The influence of the Thomson effect on the general performance, optimal performance, and optimum variables will be investigated.

## 2. Physical Model and Basic Relations

**Figure 1:**
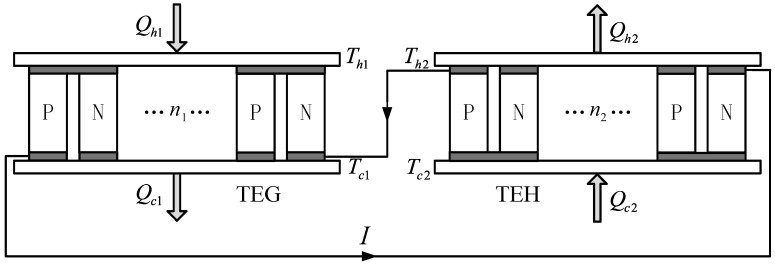
The schematic diagram of the non-equilibrium thermodynamic model for a thermoelectric generator (TEG)-thermoelectric heat pump (TEH) device.

Figure 1 shows a schematic diagram of a TEG-TEH combined device. The device consists of a TEG and a TEC connected in series; the TEG provides the input electric current (I) for the TEC. The TEG contains $n_{1}$ pairs of TE elements and the two ends of the TEG are fixed on two heat conducting and insulating connection layers. The hot and cold junction temperatures of the TEG are $T_{h1}$ and $T_{c1}$, respectively. The heat flow rates at the hot and cold junctions are $Q_{h1}$ and $Q_{c1}$, respectively. The TEH contains $n_{2}$ pairs of TE elements and the two ends of the TEH are fixed on two heat conducting and insulating connection layers. The hot and cold junction temperatures of TEH are $T_{h2}$ and $T_{c2}$, respectively. The heat flow rates at the hot and cold junctions are $Q_{h2}$ and $Q_{c2}$, respectively.

The following assumptions are made during modeling:

1. The TEG and TEH operate at steady state with constant junction temperatures;
2. The heat transfers through the TE elements are performed in a one-dimensional steady state along the TE leg;
3. The radiative and convective heat transfers around the TE elements are neglected since the modules are encapsulated adiabatically;
4. Joule heat and Thomson heat are distributed in TE elements evenly;
5. The Seebeck coefficient, electrical resistance, thermal conductivity, and the Thomson coefficient of the TE elements are dependent on temperature.

### 2.1. Energy Analysis

Based on the non-equilibrium thermodynamic theory, according to the modeling method in Reference [49], the heat absorption rate at the hot junction and the heat release rate at the cold junction of the TEG are given by Equations (1) and (2):

(1) $$Q_{h1}=n_{1}\left[\alpha_{h1}IT_{h1}+K_{1}(T_{h1}-T_{c1})-I^{2}R_{1}/2-\mu_{1}I(T_{h1}-T_{c1})/2\right]$$

(2) $$Q_{c1}=n_{1}\left[\alpha_{c1}IT_{c1}+K_{1}(T_{h1}-T_{c1})+I^{2}R_{1}/2+\mu_{1}I(T_{h1}-T_{c1})/2\right]$$

The heat release rate at the hot junction and the heat absorption rate at the cold junction of the TEH are given by Equations (3) and (4):

(3) $$Q_{h2}=n_{2}\left[\alpha_{h2}IT_{h2}-K_{2}(T_{h2}-T_{c2})+I^{2}R_{2}/2-\mu_{2}I(T_{h2}-T_{c2})/2\right]$$

(4) $$Q_{c2}=n_{2}\left[\alpha_{c2}IT_{c2}-K_{2}(T_{h2}-T_{c2})-I^{2}R_{2}/2+\mu_{2}I(T_{h2}-T_{c2})/2\right]$$

where αIT is the Peltier heat flow rate, KΔT is the Fourier heat flow rate, $I^{2}R$ is the Joule heat flow rate, and μIΔT is the Thomson heat flow rate. Parameters $\alpha_{h}$, $\alpha_{c}$, K, R, and μ are the hot and cold junction Seebeck coefficients, total thermal conductance, total electrical resistance, and Thomson coefficients, respectively; subscripts 1 and 2 denote the TEG and TEH, respectively. Parameters $\alpha_{h1}$, $\alpha_{c1}$, $K_{1}$, $R_{1}$, and $\mu_{1}$ are given by [44]

(5) $$\alpha_{h1}=\alpha_{Ph1}-\alpha_{Nh1}$$

(6) $$\alpha_{c1}=\alpha_{Pc1}-\alpha_{Nc1}$$

(7) $$K_{1}=K_{N1}+K_{P1}=\lambda_{N1}A_{N1}/l_{N1}+\lambda_{P1}A_{P1}/l_{P1}$$

(8) $$R_{1}=R_{N1}+R_{P1}=l_{N1}/\rho_{N1}A_{N1}+l_{P1}/\rho_{P1}A_{P1}$$

(9) $$\mu_{1}=\mu_{P1}-\mu_{N1}$$

where the subscripts *P* and *N* denote *P*- and *N*-type TE legs, respectively. Parameters λ, ρ, A, and l are the thermal conductivity, electrical resistance, cross-sectional area, and length of the TE leg, respectively. Parameters $\alpha_{h2}$, $\alpha_{c2}$, $K_{2}$, $R_{2}$, and $\mu_{2}$ are calculated similarly.

According to the balance of the heat flow in the system, one has

(10) $$Q_{h1}+Q_{c2}=Q_{c1}+Q_{h2}.$$

Substituting Equations (1)–(4) into Equation (10) gives the system stable electrical current:

(11) $$I_{s}=\left\{n_{1}\left[T_{h1}(\alpha_{h1}-\mu_{1})-T_{c1}(\alpha_{c1}-\mu_{1})\right]+n_{2}\left[T_{c2}(\alpha_{c2}-\mu_{2})-T_{h2}(\alpha_{h2}-\mu_{2})\right]\right\}/\left(n_{1}R_{1}+n_{2}R_{2}\right).$$

The heating load, which is the heat flow rate release to heating space at the hot junction of the TEH, is given by Equation (3). The COP can be calculated as follows:

(12) $$\begin{array}{cc} \mathrm{COP}=Q_{h2}/Q_{h1} & =n_{2}\left[\alpha_{h2}I_{s}T_{h2}-K_{2}(T_{h2}-T_{c2})+I_{s}^{2}R_{2}/2-\mu_{2}I_{s}(T_{h2}-T_{c2})/2\right]\\ & /\left\{n_{1}\left[\alpha_{h1}I_{s}T_{h1}+K_{1}(T_{h1}-T_{c1})-I_{s}^{2}R_{1}/2-\mu_{1}I_{s}(T_{h1}-T_{c1})/2\right]\right\} \end{array}$$

where the electric currents ($I_{s}$) in Equations (3) and (12) are determined by Equation (11).

The hot junction temperature of the TEH ($T_{h2}$) reaches the maximum value when the heating load is zero [22]. Therefore, substituting Equations (3) and (11) into $Q_{h2}=0$ gives the maximum heating temperature as follows:

(13) $$T_{h2,\max}=N_{Th2,\max}/D_{Th2,\max}$$

where

(14) $$\begin{array}{cc} N_{Th2,\max} & =-2K_{2}{\left(n_{1}R_{1}+n_{2}R_{2}\right)}^{2}+n_{1}\left[n_{1}R_{1}(2\alpha_{h2}-\mu_{2})+n_{2}R_{2}\mu_{2}\right]\left[(\alpha_{h1}-\mu_{1})T_{h1}-(\alpha_{c1}-\mu_{1})T_{c1}\right]\\ & \;+n_{2}\left\{n_{2}R_{2}(\alpha_{c2}-\alpha_{h2})\mu_{2}+n_{1}R_{1}\left[2\alpha_{c2}\alpha_{h2}-(\alpha_{c2}+3\alpha_{h2})\mu_{2}+2\mu_{2}^{2}\right]\right\}T_{c2}+\left|n_{1}R_{1}+n_{2}R_{2}\right|\\ & \;\times {\left\{\begin{array}{l} {\left\{n_{1}(2\alpha_{h2}-\mu_{2})\left[(\alpha_{h1}-\mu_{1})T_{h1}-(\alpha_{c1}-\mu_{1})T_{c1}\right]+n_{2}\left[2\alpha_{c2}\alpha_{h2}-(\alpha_{c2}+\alpha_{h2})\mu_{2}\right]T_{c2}\right\}}^{2}\\ -4K_{2}n_{2}\left\{n_{1}R_{1}\left[2(\alpha_{c2}-2\alpha_{h2})\alpha_{h2}-(\alpha_{c2}-3\alpha_{h2})\mu_{2}\right]-n_{2}R_{2}\left[2\alpha_{h2}^{2}-(\alpha_{c2}+\alpha_{h2})\mu_{2}\right]\right\}T_{c2}\\ -4K_{2}n_{1}\left[n_{1}R_{1}(2\alpha_{h2}-\mu_{2})+n_{2}R_{2}\mu_{2}\right]\left[(\alpha_{h1}-\mu_{1})T_{h1}-(\alpha_{c1}-\mu_{1})T_{c1}\right]+4K_{2}^{2}{\left(n_{1}R_{1}+n_{2}R_{2}\right)}^{2} \end{array}\right\}}^{0.5} \end{array}$$

(15) $$D_{Th2,\max}=2n_{2}\left[n_{2}R_{2}\alpha_{h2}+n_{1}R_{1}(2\alpha_{h2}-\mu_{2})\right](\alpha_{h2}-\mu_{2}).$$

The maximum heating temperature difference can be expressed as

(16) $$T_{d,\max}=T_{h2,\max}-T_{c2}.$$

It can be seen from Equations (14) and (15) that $T_{h2,\max}$ is a function of the junction temperatures ($T_{h1}$, $T_{c1}$ and $T_{c2}$), TE element numbers ($n_{1}$ and $n_{2}$), and physical properties.

### 2.2. Exergy Analysis

The exergy balance in the system is as follows:

(17) $$EX_{in}-EX_{out}-EX_{loss}=\Delta EX=0$$

where $EX_{in}$ is the rate of exergy input, $EX_{out}$ is the rate of exergy output, $EX_{loss}$ is the rate of exergy loss, and ΔEX is the rate of exergy gain. ΔEX is zero because of the steady state operation. The rates of exergy input, output, and loss are given by

(18) $$EX_{in}=Q_{h1}(1-T_{0}/T_{h1})-Q_{c1}(1-T_{0}/T_{c1})$$

(19) $$EX_{out}=Q_{h2}(1-T_{0}/T_{h2})-Q_{c2}(1-T_{0}/T_{c2})$$

(20) $$EX_{loss}=EX_{in}-EX_{out}$$

where $T_{0}$ is the environment temperature.

According to the second law of thermodynamics, the exergy efficiency is given as follows:

(21) $$\eta_{EX}=EX_{out}/EX_{in}=\left[Q_{h2}(1-T_{0}/T_{h2})-Q_{c2}(1-T_{0}/T_{c2})\right]/\left[Q_{h1}(1-T_{0}/T_{h1})-Q_{c1}(1-T_{0}/T_{c1})\right]$$

$\eta_{EX}$ can also be given by

(22) $$\eta_{EX}=1-EX_{loss}/EX_{in}=1-T_{0}S_{gen}/EX_{in}$$

where $S_{gen}$, which is the entropy generation rate of the system, is given by

(23) $$S_{gen}=-Q_{h1}/T_{h1}+Q_{c1}/T_{c1}+Q_{h2}/T_{h2}-Q_{c2}/T_{c2}.$$

Substituting Equations (1)–(4) into Equation (21) or Equation (22), one can obtain the exergy efficiency

(24) $$\eta_{EX}=\frac{n_{2}\left\{\begin{array}{l} \left[\alpha_{h2}I_{s}T_{h2}-K_{2}(T_{h2}-T_{c2})+I_{s}^{2}R_{2}/2-\mu_{2}I_{s}(T_{h2}-T_{c2})/2\right](1-T_{0}/T_{h2})\\ -\left[\alpha_{c2}I_{s}T_{c2}-K_{2}(T_{h2}-T_{c2})-I_{s}^{2}R_{2}/2+\mu_{2}I_{s}(T_{h2}-T_{c2})/2\right](1-T_{0}/T_{c2}) \end{array}\right\}}{n_{1}\left\{\begin{array}{l} \left[\alpha_{h1}I_{s}T_{h1}+K_{1}(T_{h1}-T_{c1})-I_{s}^{2}R_{1}/2-\mu_{1}I_{s}(T_{h1}-T_{c1})/2\right](1-T_{0}/T_{h1})\\ -\left[\alpha_{c1}I_{s}T_{c1}+K_{1}(T_{h1}-T_{c1})+I_{s}^{2}R_{1}/2+\mu_{1}I_{s}(T_{h1}-T_{c1})/2\right](1-T_{0}/T_{c1}) \end{array}\right\}}$$

where $I_{s}$ is determined by Equation (11).

Substituting $\mu_{h1}=\mu_{c1}=\mu_{h2}=\mu_{c2}=0$ into the above equations, one can obtain the physical model and basic relations without considering the Thomson effect. Simplifying the physical property parameters further—that is, setting $\alpha_{h1}=\alpha_{c1}=\alpha_{h2}=\alpha_{c2}=\alpha$, $K_{h1}=K_{c1}=K_{h2}=K_{c2}=K$, and $R_{h1}=R_{c1}=R_{h2}=R_{c2}=R$—the energy analysis becomes the results in References [21,22,23]. Therefore, a more universal non-equilibrium thermodynamic model of the TEG-TEH combined device is established in this paper since it contains results from some available literature.

## 3. Numerical Examples

The heating load of the device increases with an increase in the number of TE elements according to Equation (3), but the TE element number is finite since the initial cost and space occupied by the device also increases with an increase in the number of TE elements. Therefore, the total TE element number ($M=n_{1}+n_{2}$) is fixed in the numerical examples. To describe the allocation of the TE element between the TEG and TEH, a design variable, the ratio of TE element number is defined as $x=n_{1}/M$.

The physical property parameters and sizes of the *P*- and *N*-type TE legs of the device are assumed to be the same; namely, $\left|\alpha_{Ph1}\right|=\left|\alpha_{Nh1}\right|$, $\left|\alpha_{Pc1}\right|=\left|\alpha_{Nc1}\right|$, $\lambda_{P1}=\lambda_{N1}$, $\rho_{P1}=\rho_{N1}$, $\left|\mu_{P1}\right|=\left|\mu_{N1}\right|$, $\left|\alpha_{Ph2}\right|=\left|\alpha_{Nh2}\right|$, $\left|\alpha_{Pc2}\right|=\left|\alpha_{Nc2}\right|$, $\lambda_{P2}=\lambda_{N2}$, $\rho_{P2}=\rho_{N2}$, $\left|\mu_{P2}\right|=\left|\mu_{N2}\right|$, $l_{P1}=l_{N1}=l_{P}=l_{N2}=l$, and $A_{P1}=A_{N1}=A_{P2}=A_{N2}=A$. The detailed parameters used in the calculations are presented in Table 1.

Bismuth telluride (${\mathrm{Bi}}_{2}{\mathrm{Te}}_{3}$) is used as the TE material in the numerical examples. The temperature-dependent properties of *P*- and *N*-type TE legs can be calculated by fitting the formulas provided by Xuan et al. [50]

(25) $$\alpha_{p}=-\alpha_{N}=(22,224.0+930.6\bar{T}-0.9905{\bar{T}}^{2})\times 10^{-9}\;V\cdot K^{-1}$$

(26) $$\rho_{P}=\rho_{N}=(5,112.0+163.4\bar{T}+0.6279{\bar{T}}^{2})\times 10^{-10}\;\Omega \cdot m^{-1}$$

(27) $$\lambda_{P}=\lambda_{N}=(62,605.0-277.7\bar{T}+0.4131{\bar{T}}^{2})\times 10^{-4}\;{\mathrm{Wm}}^{-1}\cdot K^{-1}$$

where $\bar{T}$ is the average temperature of the TE element. The average temperature of the TE element of the TEG is ${\bar{T}}_{1}=\left(T_{h1}+T_{c1}\right)/2$, and the average temperature of the TE element of the TEH is ${\bar{T}}_{2}=\left(T_{h2}+T_{c2}\right)/2$. According to the second Kelvin relation [2], the Thomson coefficient is defined by

(28) μ=Tdα/dT.

Combining Equations (24) and (27) gives the Thomson coefficients of *P*- and *N*-type TE legs

(29) $$\mu_{P}=-\mu_{N}=\left(930.6\;\bar{T}-1.981\;{\bar{T}}^{2}\right)\times 10^{-9}\;V\cdot K^{-1}.$$

### 3.1. Performance Analyses

The hot junction temperatures of the TEG and TEH are set as $T_{h1}=450\;K$ and $T_{h2}=325\;K$, respectively. Variations in the performance of the device with the ratio of the TE element number are studied with and without considering the Thomson effect.

**Figure 2:**
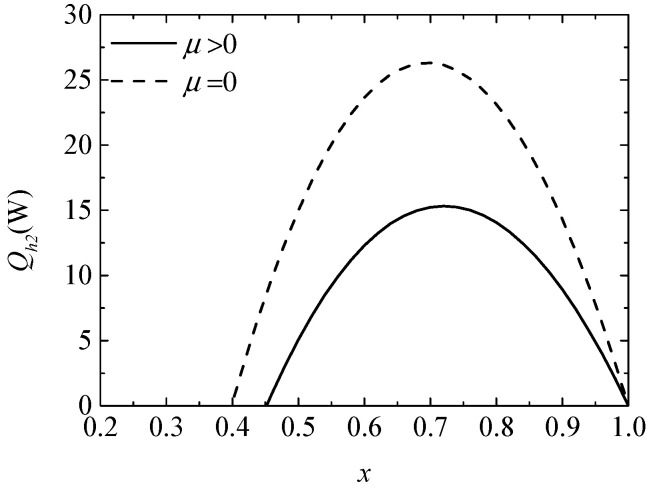
The heating load versus the ratio of the thermoelectric (TE) element number.

Figure 2 gives the variation of heating load ($Q_{h2}$) with the ratio (x) of the TE element number. In Figure 2 and the figures in the rest of this article, the solid and dashed lines represent the results with and without considering the Thomson effect, respectively. It is found that the variation of $Q_{h2}$ presents a parabola-like shape. There exist an optimum x for the maximum $Q_{h2}$ and a minimum x for the positive $Q_{h2}$. The Thomson effect decreases the $Q_{h2}$ from 14.98 W to 5.067 W when x=0.5.

**Figure 3:**
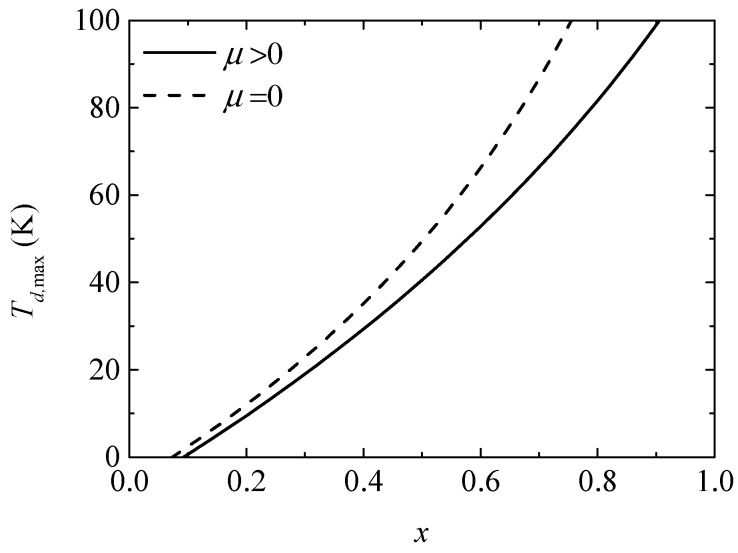
The maximum heating temperature difference versus the ratio of TE element number.

Figure 3 gives the variation of the maximum heating temperature difference ($T_{d,\max}$) with the ratio (x) of the TE element number. It is found that $T_{d,\max}$ and the slope of the curve increase with an increase in x. The Thomson effect decreases $T_{d,\max}$ from 49.39 K to 40.53 K when x=0.5.

**Figure 4:**
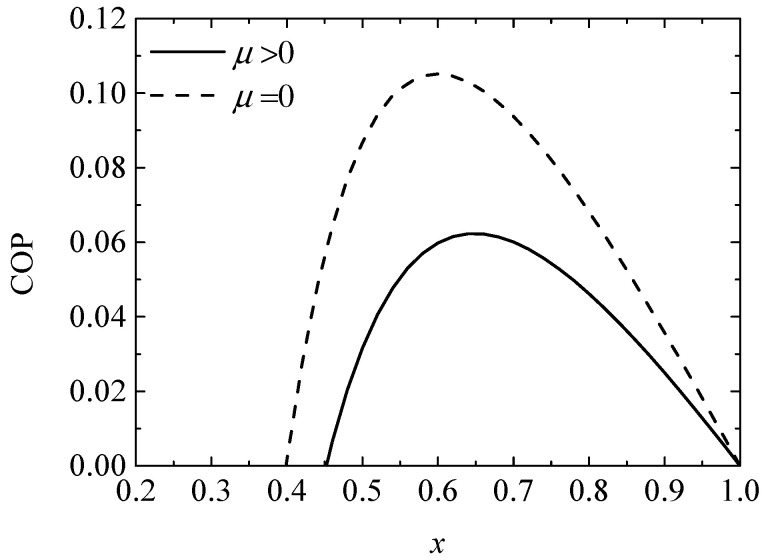
The coefficient of performance (COP) versus the ratio of TE element number.

**Figure 5:**
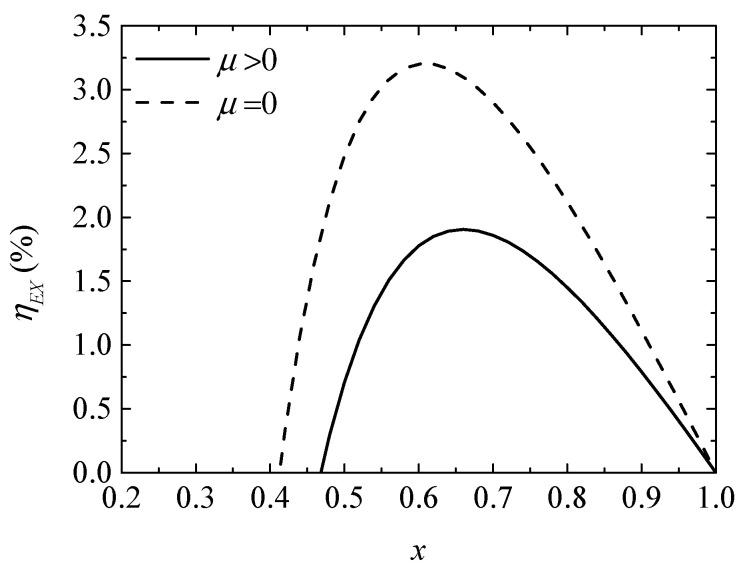
The exergy efficiency versus the ratio of TE element number.

Figure 4 and Figure 5 give the variations of the COP and exergy efficiency ($\eta_{EX}$) with the ratio (x) of the TE element number, respectively. It is found that variations of COP and $\eta_{EX}$ present parabola-like shapes. Two optima x exist for the maximum COP and maximum $\eta_{EX}$, respectively, and two minima x exist for the positive COP and positive $\eta_{EX}$. The Thomson effect decreases the COP and $\eta_{EX}$ from 0.08683 and 2.480% to 0.03162 and 0.7073%, respectively, when x=0.5.

The optimum current ($I_{opt}$) can be calculated through Equation (11) for a given optimum x. The $I_{opt}$ for maximum $Q_{h2}$, maximum COP, and maximum $\eta_{EX}$ considering the Thomson effect are 0.7172 A, 0.6321 A, and 0.6464 A, respectively. Those without considering the Thomson effect are 0.8780 A, 0.7252 A, and 0.7399 A, respectively. The electrical current reaches a maximum of 1.310 A in a short circuit condition. It can be seen that the maximum currents allowed by commercial TE modules can meet the requirements of optimal performance.

### 3.2. Performance Optimizations

According to the performance analyses, there are optimum structural designs that maximize the heating load, COP, and exergy efficiency of a TEG-TEH combined device, so performance optimizations are necessary. For optimizing the allocation of the TE element number for maximum heating load, maximum COP, and maximum exergy efficiency, the nomenclatures of the optimal performance and optimum x ($x_{opt}$) obtained are listed in Table 2.

The hot junction temperatures of the TEG and TEH, which determine the working surroundings, are important factors. Therefore, variations in the optimal performance and $x_{opt}$ with hot junction temperatures of a TEG and TEH are studied with and without considering the Thomson effect in the performance optimizations.

The allocation of TE elements is optimized by solving non-linear integer programming since the TE element numbers ($n_{1}$ and $n_{2}$) are integers. The curves of the optimal performances and $x_{opt}$ versus the junction temperatures, which are discrete, are plotted as continuous curves to reflect the tendencies better. It has been proven by comparison that the difference between the discrete and continuous curves is small.

#### 3.2.1. Effects of the TEG Hot Junction Temperature

**Figure 6:**
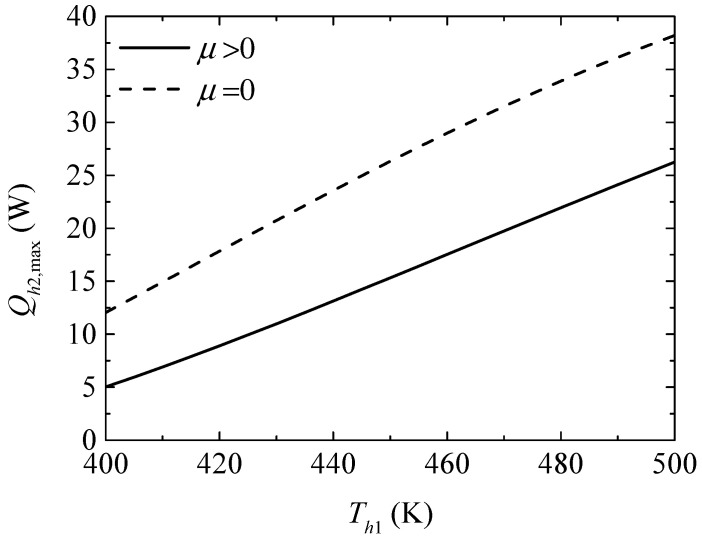
The maximum heating load versus the TEG hot junction temperature.

**Figure 7:**
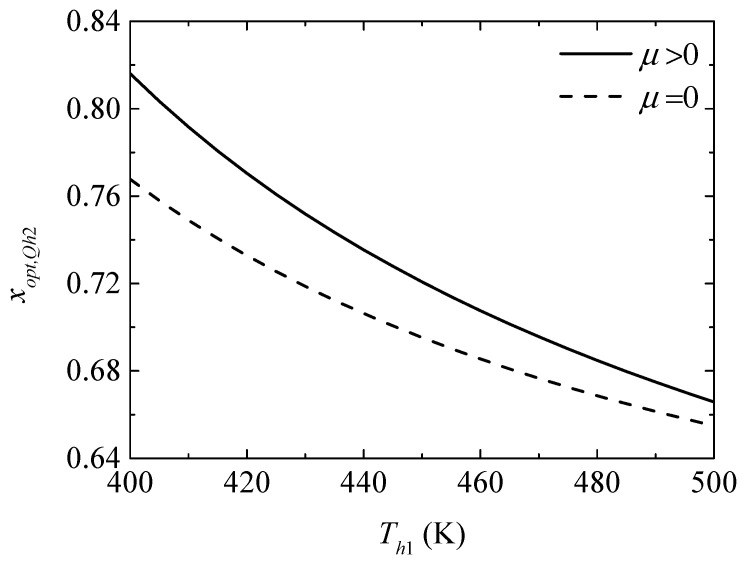
The optimum ratio of the TE element number for maximum heating load versus the TEG hot junction temperature.

Figure 6 gives the effect of the TEG hot junction temperature ($T_{h1}$) on the maximum heating loads ($Q_{h2,\max}$ and $Q_{h2,\max}^{\prime }$) with and without considering the Thomson effect when the TEH hot junction temperature is set at $T_{h2}=325\;K$. Figure 7 gives the effect of $T_{h1}$ on the optimum ratios ($x_{opt,Qh2}$ and $x_{opt,Qh2}^{\prime }$) of the TE element number at the maximum $Q_{h2}$. It can be seen from Figure 6 that $Q_{h2,\max}$ and $Q_{h2,\max}^{\prime }$ increase with increasing $T_{h1}$. The Thomson effect decreases the maximum $Q_{h2}$ at different $T_{h1}$. It can be seen from Figure 7 that $x_{opt,Qh2}$, $x_{opt,Qh2}^{\prime }$, and the slopes of the curves decrease with increasing $T_{h1}$. The Thomson effect increases the optimum x at the maximum $Q_{h2}$ at different $T_{h1}$.

**Figure 8:**
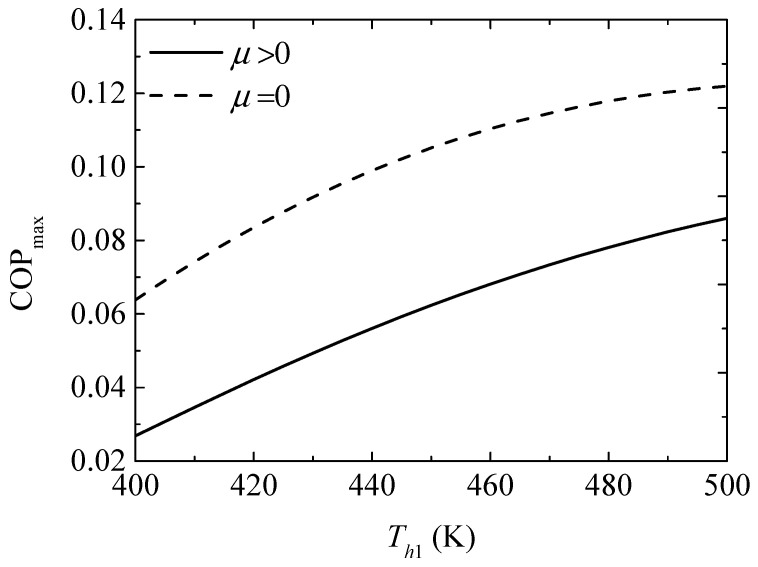
The maximum COP versus the TEG hot junction temperature.

**Figure 9:**
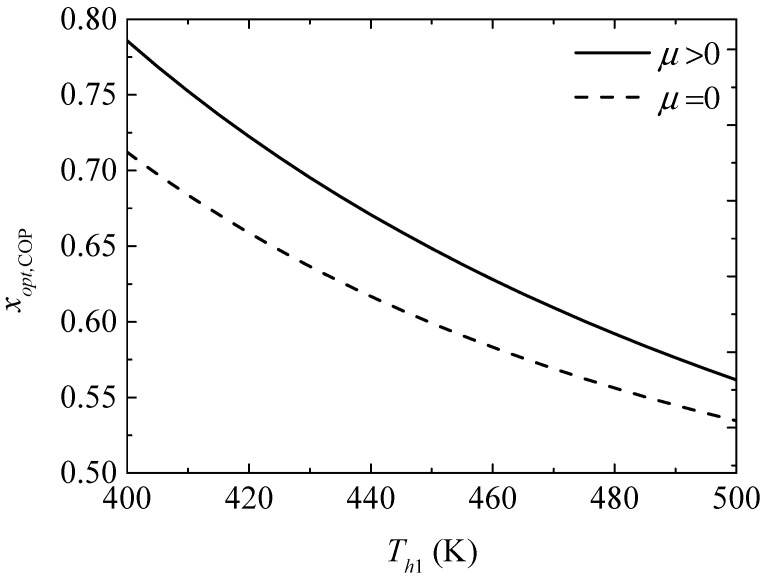
The optimum ratio of the TE element number for the maximum COP versus the TEG hot junction temperature.

Figure 8 gives the effect of the TEG hot junction temperature ($T_{h1}$) on the maximum COPs (${\mathrm{COP}}_{\max}$ and ${\mathrm{COP}}_{\max}^{\prime }$) with and without considering the Thomson effect when the TEH hot junction temperature is set at $T_{h2}=325\;K$. Figure 9 gives the effect of $T_{h1}$ on the optimum ratios ($x_{opt,\mathrm{COP}}$ and $x_{opt,\mathrm{COP}}^{\prime }$) of the TE element number at the maximum COP. It can be seen from Figure 8 that ${\mathrm{COP}}_{\max}$ and ${\mathrm{COP}}_{\max}^{\prime }$ increase and the slopes of the curves decrease with increasing $T_{h1}$; a higher $T_{h1}$ leads to a gentle variation of ${\mathrm{COP}}_{\max}^{\prime }$. That is, the maximum COP can be increased effectively by means of increasing $T_{h1}$ at lower $T_{h1}$, but the method is not effective at higher $T_{h1}$ without considering the Thomson effect. The Thomson effect decreases the maximum COP at different $T_{h1}$. It can be seen from Figure 9 that $x_{opt,\mathrm{COP}}$, $x_{opt,\mathrm{COP}}^{\prime }$, and the slopes of the curves decrease with increasing $T_{h1}$. The Thomson effect increases the optimum x at the maximum COP at different $T_{h1}$.

**Figure 10:**
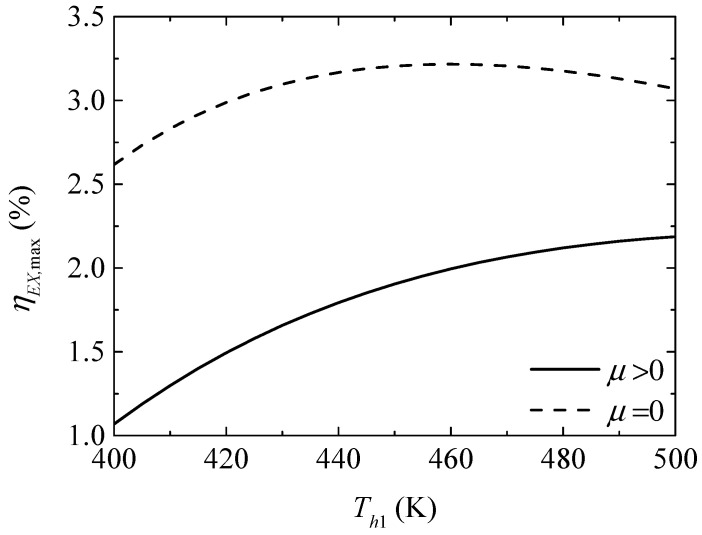
The maximum exergy efficiency versus the TEG hot junction temperature.

**Figure 11:**
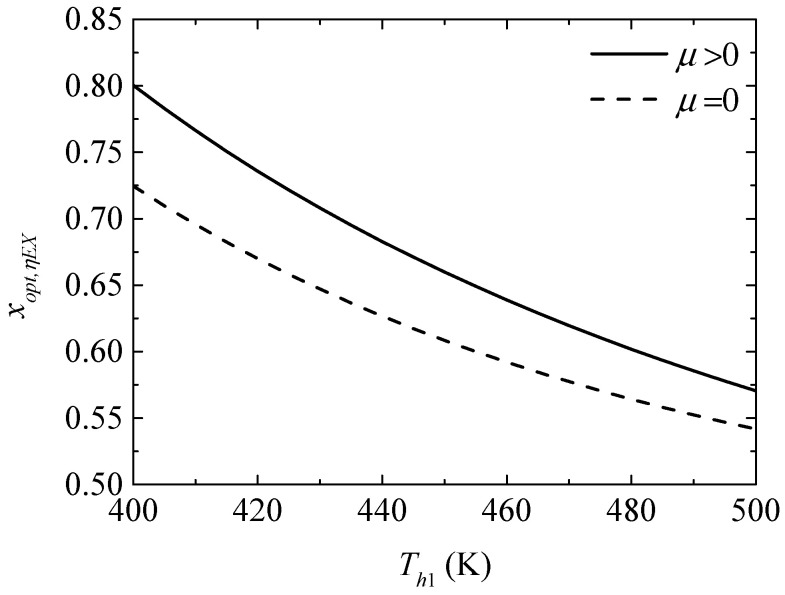
The optimum ratio of the TE element number for the maximum exergy efficiency versus the TEG hot junction temperature.

Figure 10 gives the effect of the TEG hot junction temperature ($T_{h1}$) on the maximum exergy efficiencies ($\eta_{EX,\max}$ and $\eta_{EX,\max}^{\prime }$) with and without considering the Thomson effect when the TEH hot junction temperature is set at $T_{h2}=325\;K$. Figure 11 gives the effect of $T_{h1}$ on the optimum ratios ($x_{opt,\eta EX}$ and $x_{opt,\eta EX}^{\prime }$) of the TE element number at the maximum $\eta_{EX}$. It can be seen from Figure 10 that $\eta_{EX,\max}^{\prime }$ increases at first and then decreases with increasing $T_{h1}$; $\eta_{EX,}{}_{\max}$ increases and the slope of the curve decreases with increasing $T_{h1}$; a higher $T_{h1}$ leads to a gentle variation of $\eta_{EX,\max}$. That is, the maximum $\eta_{EX}$ can be increased effectively by means of increasing $T_{h1}$ at a lower $T_{h1}$, but the method is not effective considering the Thomson effect and even counterproductive without considering the Thomson effect at higher $T_{h1}$. The Thomson effect decreases the maximum $\eta_{EX}$ at different $T_{h1}$. It can be seen from Figure 11 that $x_{opt,\eta EX}$, $x_{opt,\eta EX}^{\prime }$, and the slopes of the curves decrease with increasing $T_{h1}$. The Thomson effect increases the optimum x at the maximum $\eta_{EX}$ at different $T_{h1}$.

It can be concluded from Figure 7, Figure 9 and Figure 11 that the Thomson effect increases the optimum x at different $T_{h1}$. That is, due to considering the Thomson effect, more TE elements should be allocated to TEGs appropriately at different $T_{h1}$ to ensure the device is designed at the optimal performance. The optimum ratios of TE element number decrease with increasing $T_{h1}$. That is, as $T_{h1}$ increases, more TE elements should be appropriately allocated to the TEH to ensure that the device is designed at the optimal performance.

#### 3.2.2. Effects of the TEH Hot Junction Temperature

**Figure 12:**
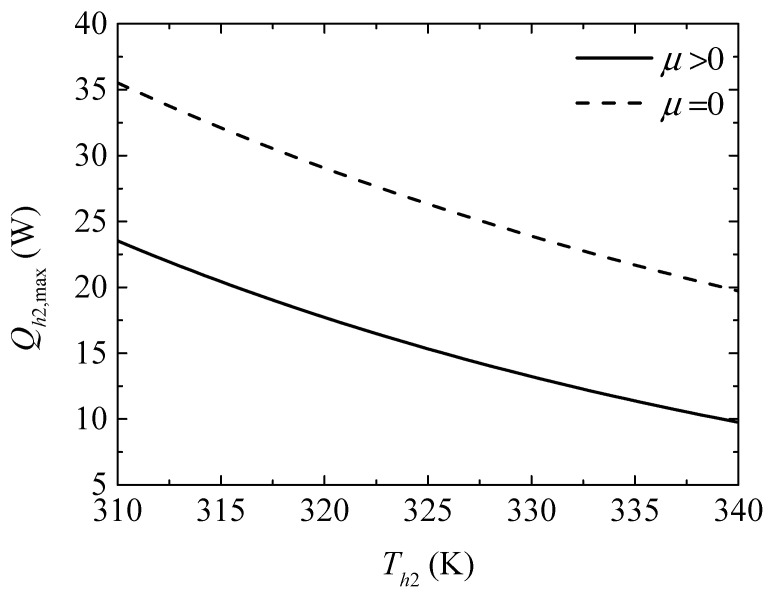
The maximum heating load versus the TEH hot junction temperature.

**Figure 13:**
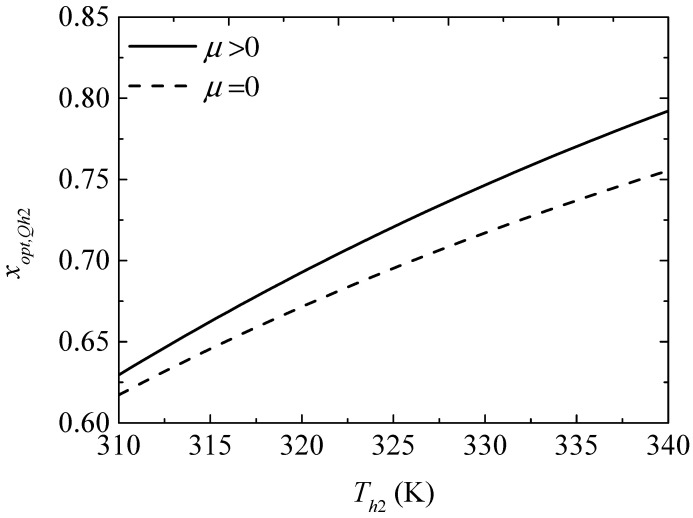
The optimum ratio of TE element number for the maximum heating load versus the TEH hot junction temperature.

Figure 12 gives the effect of the TEH hot junction temperature ($T_{h2}$) on the maximum heating powers ($Q_{h2}{}_{,\max}$ and $Q_{h2,\max}^{\prime }$) with and without considering the Thomson effect when the TEG hot junction temperature is set at $T_{h1}=450\;K$. Figure 13 gives the effect of $T_{h2}$ on the optimum ratios ($x_{opt,Qh2}$ and $x_{opt,Qh2}^{\prime }$) of the TE element number at the maximum $Q_{h2}$. It can be seen from Figure 12 that $Q_{h2,\max}$, $Q_{h2,\max}^{\prime }$, and the slopes of the curves decrease with increasing $T_{h2}$. The Thomson effect decreases the maximum $Q_{h2}$ at different $T_{h2}$. It can be seen from Figure 13 that $x_{opt,Qh2}$ and $x_{opt,Qh2}^{\prime }$ increase and the slopes of the curves decrease with increasing $T_{h2}$. The Thomson effect increases the optimum x at the maximum $Q_{h2}$ at different $T_{h2}$.

**Figure 14:**
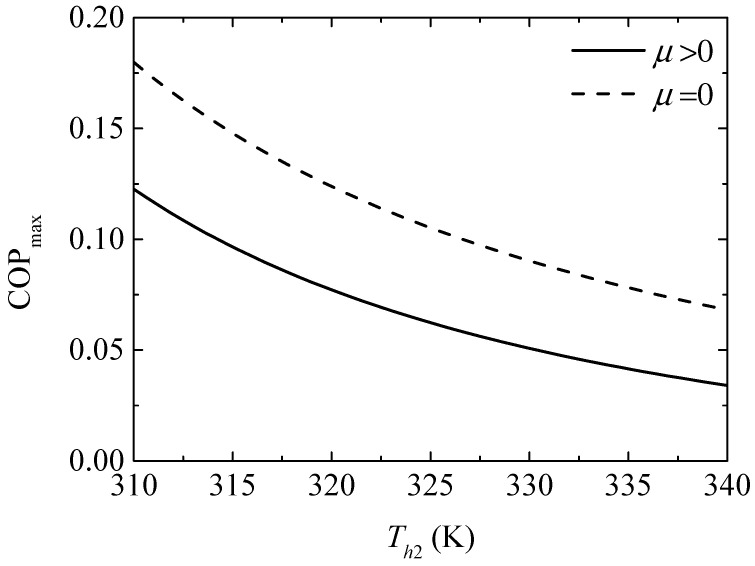
The maximum COP versus the TEH hot junction temperature.

**Figure 15:**
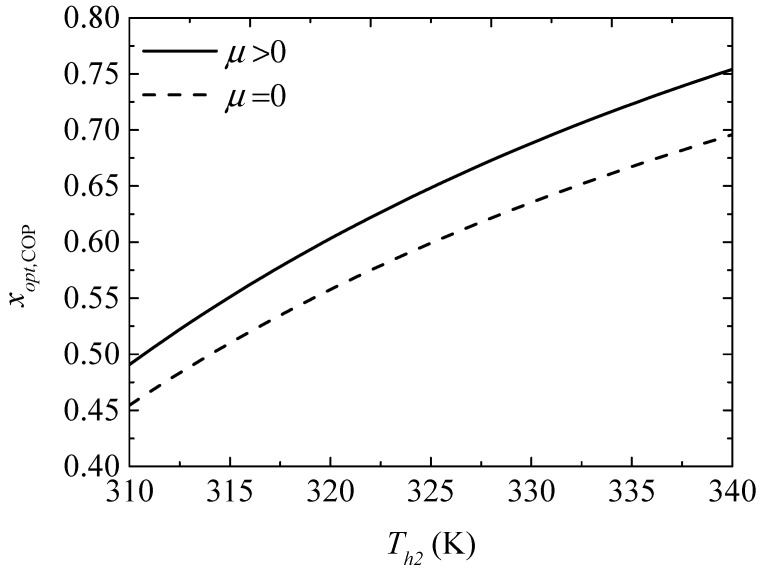
The optimum ratio of the TE element number for the maximum COP versus the TEH hot junction temperature.

Figure 14 gives the effect of the TEH hot junction temperature ($T_{h2}$) on the maximum COPs (${\mathrm{COP}}_{\max}$ and ${\mathrm{COP}}_{\max}^{\prime }$) with and without considering the Thomson effect when the TEG hot junction temperature is set at $T_{h1}=450\;K$. Figure 15 gives the effect of $T_{h2}$ on the optimum ratios ($x_{opt,\mathrm{COP}}$ and $x_{opt,\mathrm{COP}}^{\prime }$) of the TE element number at the maximum COP. It can be seen from Figure 14 that ${\mathrm{COP}}_{\max}$, ${\mathrm{COP}}_{\max}^{\prime }$, and the slopes of the curves decrease with increasing $T_{h2}$. The Thomson effect decreases the maximum COP at different $T_{h2}$. It can be seen from Figure 15 that $x_{opt,\mathrm{COP}}$ and $x_{opt,\mathrm{COP}}^{\prime }$ increase and the slopes of the curves decrease with increasing $T_{h2}$. The Thomson effect increases the optimum x at the maximum COP at different $T_{h2}$.

**Figure 16:**
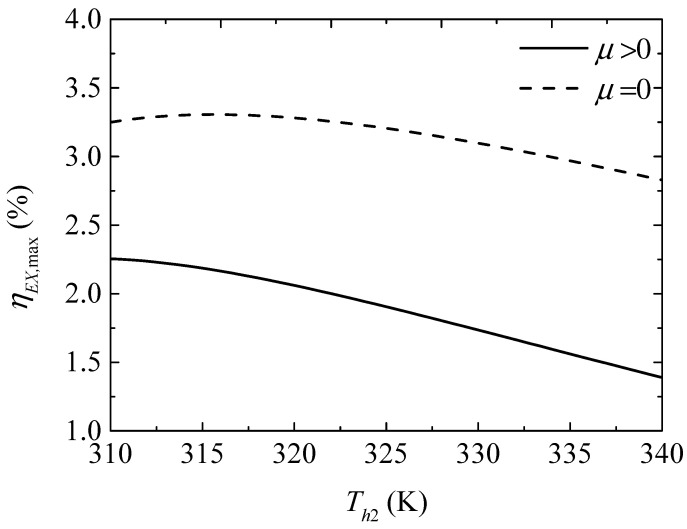
The maximum exergy efficiency versus the TEH hot junction temperature.

**Figure 17:**
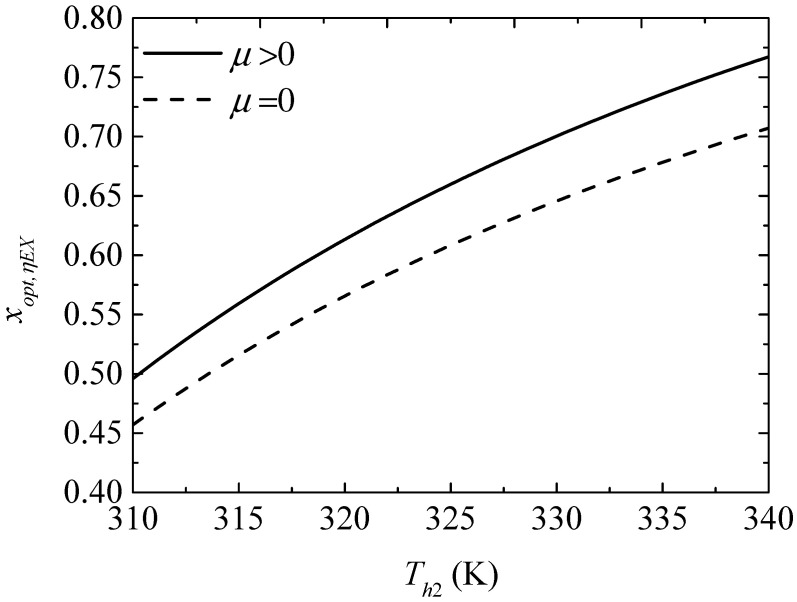
The optimum ratio of the TE element number for the maximum exergy efficiency versus the TEH hot junction temperature.

Figure 16 gives the effect of the TEH hot junction temperature ($T_{h2}$) on the maximum exergy efficiencies ($\eta_{EX,\max}$ and $\eta_{EX,\max}^{\prime }$) with and without considering the Thomson effect when the TEG hot junction temperature is set at $T_{h1}=450\;K$. Figure 17 gives the effect of $T_{h2}$ on the optimum ratios ($x_{opt,\eta EX}$ and $x_{opt,\eta EX}^{\prime }$) of the TE element number at the maximum $\eta_{EX}$. It can be seen from Figure 16 that $\eta_{EX,\max}$ and $\eta_{EX,\max}^{\prime }$ increase at first and then decrease with increasing $T_{h2}$. The Thomson effect decreases the maximum $\eta_{EX}$ at different $T_{h2}$. It can be seen from Figure 17 that $x_{opt,\eta EX}$ and $x_{opt,\eta EX}^{\prime }$ increase and the slopes of the curves decrease with increasing $T_{h2}$. The Thomson effect increases the optimum x at the maximum $\eta_{EX}$ at different $T_{h2}$.

It can be concluded from Figure 13, Figure 15 and Figure 17 that the Thomson effect increases the optimum x at different $T_{h2}$. That is, due to considering the Thomson effect, more TE elements should be allocated to the TEG appropriately at different $T_{h2}$ to ensure that the device is designed at the optimal performance. The optimum ratios of the TE element number increase with increasing $T_{h2}$. That is, as $T_{h2}$ increases, more TE elements should be allocated to the TEG appropriately to ensure that the device is designed at the optimal performance.

Comparing Figure 9 with Figure 11 and Figure 15 with Figure 17, it is found that the optimum x at the maximum $\eta_{EX}$ is greater than that at the maximum COP. That is, more TE elements should be appropriately allocated to the TEG for maximum $\eta_{EX}$ compared to that for the maximum COP.

#### 3.2.3. The Ranges of Optimal Performance and Optimum Variables

A higher $Q_{h2}$ leads to a more efficient heating effect, while a higher COP or $\eta_{EX}$ leads to more economical performance. So the TEG-TEH combined device should be designed with the best heating effect and the best economical performance (i.e., the optimal performance ranges that include the optimal ranges of $Q_{h2}$, COP, and $\eta_{EX}$).

Taking COP as an economical index, the optimal ranges of $Q_{h2}$, COP, and $\eta_{EX}$—considering the Thomson effect—are $\left[Q_{h2,\mathrm{COP}},\;Q_{h2,\max}\right]$, $\left[{\mathrm{COP}}_{Qh2},\;{\mathrm{COP}}_{\max}\right]$, and $\left[\eta_{EX,Qc2},\eta_{EX,\max}\right]$, respectively. Those without considering the Thomson effect are $\left[Q_{h2,\mathrm{COP}}^{\prime },\;Q_{h2,\max}^{\prime }\right]$, $\left[{\mathrm{COP}}_{Qh2}^{\prime },\;{\mathrm{COP}}_{\max}^{\prime }\right]$, and $\left[\eta_{EX,Qh2}^{\prime },\eta_{EX,\max}^{\prime }\right]$, respectively. Table 3 lists the optimization results for the maximum $Q_{h2}$, maximum COP, and maximum $\eta_{EX}$ when $T_{h1}=450\;K$ and $T_{h2}=325\;K$ are set. It is found that the Thomson effect narrows the optimal ranges of $Q_{h2}$, COP, and $\eta_{EX}$.

It can be seen from Table 3 and Figure 9, Figure 11, Figure 15 and Figure 17 that the optimum x at the maximum $Q_{h2}$ is greater than that at the maximum COP. So, x should be set within the optimum x ranges, which are $\left[x_{opt,\mathrm{COP}},x_{opt,Qh2}\right]$ and $\left[x_{opt,\mathrm{COP}}^{\prime },x_{opt,Qh2}^{\prime }\right]$ with and without considering the Thomson effect, respectively, to ensure the device is designed within the optimal performance ranges. It can be concluded that the Thomson effect narrows the optimum x range.

The Thomson effect narrows the ranges of the optimal performance and optimum x range and the optimal variables when $\eta_{EX}$ is taken as the economical index.

## 4. Conclusions

The influence of the Thomson effect on general performance, optimal performance, and optimum variables are studied in the performance analyses and optimization. The main conclusions are as follows:

(1) The Thomson effect decreases the general performance of the TEG-TEH combined device. It decreases the heating load, maximum heating temperature difference, COP, and exergy efficiency from 24.11 W, 53.22 K, 0.1329, and 3.656% to 12.47 W, 43.64 K, 0.07459, and 2.035% when the TE elements are allocated equally between the TEG and TEH, the hot and cold junction temperatures of the TEG are set at 450 K and 305 K, respectively, and those of the TEH are set at 325 K and 295 K, respectively.
(2) The Thomson effect decreases the optimal performance of the TEG-TEH combined device. It decreases the maximum heating load, maximum COP, and maximum exergy efficiency from 26.33 W, 0.1052 W, and 3.205% to 15.33 W, 0.06234 W, and 1.905% when the hot and cold junction temperatures of the TEG are set at 450 K and 305 K, respectively, and those of TEH are set at 325 K and 295 K, respectively.
(3) The Thomson effect narrows the optimal performance ranges and optimum ratio of the TE element number. More TE elements should be appropriately allocated to the TEG to ensure that the TEG-TEH combined device is designed at the optimal performance when the Thomson effect is considered.
(4) In designing TEG-TEH combined devices, more TE elements should be allocated to the TEG for the maximum exergy efficiency compared to the maximum COP.
(5) More TE elements should be allocated to the TEH to ensure that the TEG-TEH combined device is designed at the optimal performance with the increase in TEG hot junction temperature or decrease in TEH hot junction temperature.

Bismuth telluride was used as the TE material in the numerical examples in this paper. TE materials with highly efficient compositions including half-Heuslers [51] and tellurides [52,53] might be used in numerical examples in the next step.

## Figures and Tables

**Table 1:** Settings and parameter ranges.

l (m)	A ($m^{2}$)	M	$T_{0}$ (K)	$T_{h1}$ (K)	$T_{c1}$ (K)	$T_{h2}$ (K)	$T_{c2}$ (K)	x
2 × 10^−3^	1 × 10^−6^	1000	298	[400,500]	310	[310,340]	290	(0, 1)

**Table 2:** Nomenclatures of the optimal performance and the corresponding optimum variables.

Optimal Objective	With or Without Considering the Thomson Effect	$x_{opt}$	$Q_{h2}$	COP	$\eta_{EX}$
maximum heating load	μ>0	$x_{opt,Qh2}$	$Q_{h2}{}_{,\max}$	${\mathrm{COP}}_{Qh2}$	$\eta_{EX,}{}_{Qh2}$
	μ=0	$x_{opt,Qh2}^{\prime }$	$Q_{h2，\max}^{\prime }$	${\mathrm{COP}}_{Qh2}^{\prime }$	$\eta_{EX,Qh2}^{\prime }$
maximum COP	μ>0	$x_{opt,COP}$	$Q_{h2}{}_{,COP}$	${\mathrm{COP}}_{\max}$	$\eta_{EX,}{}_{COP}$
	μ=0	$x_{opt,COP}^{\prime }$	$Q_{h2,COP}^{\prime }$	${\mathrm{COP}}_{\max}^{\prime }$	$\eta_{EX,COP}^{\prime }$
optimal exergy efficiency	μ>0	$x_{opt,\eta EX}$	$Q_{h2}{}_{,\eta EX}$	${\mathrm{COP}}_{\eta EX}$	$\eta_{EX,}{}_{\max}$
	μ=0	$x_{opt,\eta EX}^{\prime }$	$Q_{h2,\eta EX}^{\prime }$	${\mathrm{COP}}_{\eta EX}^{\prime }$	$\eta_{EX,max}^{\prime }$

**Table 3:** Optimal performances and the corresponding optimum variables with $T_{h1}=450\;K$ and $T_{h2}=325\;K$.

Optimal Objective	With or Without Considering the Thomson Effect	$x_{opt}$	$Q_{h2}$ (W)	COP (%)	$\eta_{EX}$ (%)
maximum heating power	μ>0	0.7210	15.33	5.803	1.804
	μ=0	0.6950	26.33	9.473	2.929
maximum COP	μ>0	0.6480	14.24	6.234	1.900
	μ=0	0.5990	23.59	10.52	3.201
optimal exergy efficiency	μ>0	0.6600	14.57	6.221	1.905
	μ=0	0.6080	24.08	10.50	3.205

## Nomenclature

*A*	cross sectional area of TE leg (m^2^)
EX	rate of exergy (W)
*I*	system stable current (A)
*K*	thermal conductance (WK^−1^)
*l*	length of thermoelectric leg (m)
*M*	total thermoelectric elements number
n	thermoelectric elements number
*Q*	heat flow rate (W)
*R*	electrical resistance (Ω)
$S_{gen}$	entropy generation rate (WK^−1^)
*T*	temperature (K)
*x*	ratio of thermoelectric element number

## Greek Symbols

*α*	Seebeck coefficient (VK^−1^)
η	efficiency
*μ*	Thomson coefficient (VK^−1^)
*ρ*	electrical resistivity (Ωm)
*λ*	thermal conductivity (Wm^−1^K^−1^)

## Superscript

—	mean

## Subscripts

0	environment
1	TEG
2	TEC
*c*	cold junction
*h*	hot junction
in	input
loss	exergy loss
max	maximum
*N*	*N*-type thermoelectric leg
*opt*	optimum
out	output
*P*	*P*-type thermoelectric leg
*s*	practical solution of operating electrical current equation
COP	coefficient of performance
TE	thermoelectric
TEC	thermoelectric cooler
TEG	thermoelectric generator
TEH	thermoelectric heat pump
